# Expression of TLR4 in Non-Small Cell Lung Cancer Is Associated with PD-L1 and Poor Prognosis in Patients Receiving Pulmonectomy

**DOI:** 10.3389/fimmu.2017.00456

**Published:** 2017-04-21

**Authors:** Kaiyuan Wang, Jian Wang, Feng Wei, Ning Zhao, Fan Yang, Xiubao Ren

**Affiliations:** ^1^Department of Immunology, Tianjin Medical University Cancer Institute and Hospital, Tianjin, China; ^2^Department of Anesthesiology, Tianjin Medical University Cancer Institute and Hospital, Tianjin, China; ^3^National Clinical Research Center for Cancer, Tianjin, China; ^4^Tianjin Key Laboratory of Cancer Immunology and Biotherapy, Tianjin, China; ^5^Department of Biotherapy, Tianjin Medical University Cancer Institute and Hospital, Tianjin, China

**Keywords:** toll-like receptor 4, non-small cell lung cancer, programmed cell death ligand 1, soluble toll-like receptor 4, tumor microenvironment

## Abstract

Currently, the effect of inflammation on tumorigenesis and progression has been widely noted. As a member of pattern recognition receptors, toll-like receptor 4 (TLR4) plays a pivotal role in tumor immune microenvironment and has been increasingly investigated. In the present study, we evaluated TLR4 expression and its association with programmed cell death ligand 1 (PD-L1) in non-small cell lung cancer (NSCLC) tissues and assessed the predicting value of TLR4 on postoperative outcome. A total of 126 NSCLC patients receiving complete pulmonary resection and systematic lymph node dissection between April 2008 and August 2014 were enrolled. All the patients had integrated clinicopathological records and follow-up data. TLR4 and PD-L1 expression on NSCLC samples were determined by immunohistochemistry, and serum soluble TLR4 (sTLR4) levels were measured by enzyme-linked immunosorbent assay. Results showed that TLR4 expression level in cancer tissue was significantly higher than that in para-cancer tissue. Elevated TLR4 expression was significantly associated with histological type (adenocarcinoma higher than squamous cell carcinoma, *P* = 0.041), increased clinical TNM stage (*P* < 0.001), and presence of lymphatic invasion (*P* < 0.001). Besides, TLR4 expression level in cancer samples was inversely correlated with serum sTLR4 level in patients with early-stage NSCLC (*r* = −0.485, *P* = 0.003). TLR4 expression level was also positively correlated with the PD-L1 expression level (*r* = 0.545, *P* < 0.0001). Multivariate analysis showed that expression level of TLR4 was an independent prognostic factor and TLR4 overexpression indicated a poor overall survival and disease-free survival. Taken together, we conclude that expression of TLR4 in lung cancer is associated with PD-L1 and could predict the outcome of patients with NSCLC receiving pulmonary resection for cancer.

## Introduction

Currently, the role of inflammation in tumorigenesis and progression has become evident, and it is generally accepted that an inflammatory microenvironment is an essential component of all tumors (1). Especially, cell senescence and most solid malignancies in older individuals and even old age are postulated to be tumor promoters acting through inflammatory mechanisms (2, 3).

As members of pattern recognition receptors, toll-like receptors (TLRs) play a crucial role in inflammation and innate immune function by recognizing pathogen-associated molecular patterns (PAMPs) such as lipopolysaccharide (LPS), lipopeptides, dsRNA, and bacterial DNA (4, 5). As the first identified human Toll homolog, activation of toll-like receptor 4 (TLR4) in immune cells could trigger production of pro-inflammatory cytokines and other mediators against pathogens (6). Except for immune cells, endothelial and various cancer cells also express TLR4, and chronic stimulation of TLR4 could promote tumor initiation, growth, and tumor immune escape and induce chemoresistance through pro-inflammatory responses and antiapoptotic signals. Consequently, the inverse correlation of cancer expressed TLR4, and patients’ outcome has been reported (7, 8).

Our previous study showed that low serum soluble TLR4 (sTLR4) could predict poor survival of early-stage non-small cell lung cancer (NSCLC) patients who received surgical resection (9). Other studies reported that levels of TLR4 were strongly expressed in lung cancer tissue and had a positive correlation with the malignancy of lung cancer (10, 11). However, whether expression of TLR4 in NSCLC cancer tissue could be identified as a prognostic marker needs to be investigated.

Programmed cell death ligand 1 (PD-L1), also known as B7-H1, is a new member of B7 family. Tumor-associated PD-L1 has been shown to suppress tumor-specific T cell-mediated immunity by inducing T cell apoptosis, inhibiting cytokine production, and impairing the cytotoxicity of activated T cells (12–14). Recent studies showed that stimulation of TLR4 could induce PD-L1 dynamic expression on DCs, macrophages, colonic stromal cells, and cancer cells (15–18). Therefore, it is hypothesized that the expression level of TLR4 is associated with PD-L1 in cancer cells.

In the present study, we assayed the expression level and association of TLR4 and PD-L1 in lung cancer tissues and evaluated the predicting role of TLR4 in cancer cells on postoperative survival in NSCLC patients receiving pulmonary lobectomy. On the base of our former discovery, we also further explored the relationship between serum sTLR4 level and cancer tissue expressed TLR4 level.

## Materials and Methods

### Patients

A total of 126 lung cancer patients receiving complete pulmonary resection and systematic lymph node dissection from Department of Lung Carcinoma, Tianjin Medical University Cancer Institute and Hospital (TMUCIH), Tianjin, China, between April 2008 and August 2014 were enrolled. All patients were pathologically diagnosed for the first time as NSCLC during the enrollment period and classified into TNM stages according to the most recent International Association for the Study of Lung Cancer TNM classification system (19). All the patients received preoperative 18F-fluorodeoxyglucose positron emission tomography combined with computed tomography (18F-FDG-PET/CT) and the maximum standardized uptake value (SUVmax) was recorded.

Each enrolled patient had a complete clinicopathological and follow-up data. The postoperative treatment for patients was strictly ground on the National Comprehensive Cancer Network Clinical Practice Guideline in NSCLC. Exclusion criteria included patients with previous or simultaneous cancers; patients receiving preoperative anticancer treatment such as neo-adjuvant chemotherapy, radiotherapy, or biotherapy; and patients died from non-cancer disease or died within 1 month after surgery.

All patients were followed up until August 30, 2015. Patients who were still alive after the last follow-up were censored in the retrospective study. Overall survival (OS) was defined as the period between the time of surgery to death or to the last follow-up. Disease-free survival (DFS) time was interval between the time of surgery and the time when recurrence was diagnosed or to the time of the last date of follow-up.

### Immunohistochemistry (IHC)

Primary antibodies used for IHC of lung carcinoma tissues included monoclonal mouse anti-TLR4 antibody (1:100, Abcam, Cambridge, MA, USA, clone 76B357.1, cat No. ab22048), and polyclonal rabbit anti-PD-L1 antibody (1:400, Proteintech, Chicago, IL, USA, cat No. 17952-1-AP). The 60 samples of normal lung at 10 cm from the cancer were taken as para-carcinoma controls. Paraffin-embedded samples were baked for 1 h at 70°C and de-paraffinized. Tissue was then fixed with formaldehyde and blocked with 5% serum for 1 h; antigen retrieval was by heat mediation in citrate buffer (10 mM, pH 6). Endogenous peroxidase activity was inhibited with 0.3% hydrogen peroxide for 20 min. The tissues were then incubated with primary antibodies at prescribed dilutions overnight at 4°C. After rinsing with PBS, an undiluted horseradish peroxidase (HRP)-conjugated goat anti-mouse or goat anti-rabbit IgG polyclonal was added to the tissue as the secondary antibody for 30 min at 37°C. The slides were washed and incubated with 3,3′-diaminobenzidine (DAB) chromogen substrate (ZSGB-BIO, China). The nucleus was stained by hematoxylin (Sigma-Aldrich, Switzerland). Finally, cover the tissue sections with a glass coverslip and assess by light microscope.

Tumor sections were evaluated in consensus viewpoints by two pathologists who were blinded to the identity of the tissue. Positive samples of TLR4 and PD-L1 were defined as those showing membranous or cytoplasmic staining of tumor cells. Briefly, IHC score was acquired by the semi-quantitative method that taking staining intensity and proportion of cell stained together into account. For TLR4, the staining intensity was scored into four categories according to the color of immune reaction: negative, 0; light brown, 1; brown, 2; and dark brown, 3. The proportion of positively stained cells was determined and scored as: ≤5%, 0; 6–25%, 1; 26–50%, 2; 51–75%, 3; and 76–100%, 4. The expression level of TLR4 was obtained by multiplying the intensity and proportion score. And a final score of 0–2 was considered as negative expression (−, noted as 0); 3–4: weak positive expression (+, noted as 1); 6–8: moderate positive expression (++, noted as 2); and 9–12 was strong positive expression (+++, noted as 3) (10). For PD-L1, based on previous studies, the samples with stained tumor cells < 5% was defined as negative (−, scored as 0) (20, 21). For PD-L1 positive ≥5% samples, the expression level were further classified into weak positive (+, noted as 1), moderate positive (++, noted as 2), and strong positive expression (+++, noted as 3) according to staining intensity from light brown to brown and dark brown, correspondingly (22). Negative and weak positive expression were defined as low expression, and moderate and strong positive expression were defined as high expression for TLR4 and PD-L1.

### Serum sTLR4 Level by Enzyme-Linked Immunosorbent Assay (ELISA)

Serum samples from 52 enrolled NSCLC patients between January 2012 and August 2014 were obtained from Cancer Biobank of TMUCIH. Blood samples were collected prior to any anticancer therapy in non-heparinized tubes and serum samples were prepared by centrifugation and stored at −80°C until tested. The concentrations of sTLR4 were assayed by ELISA (Abnova, USA) according to manufacturer’s instructions, as described in our previous research (9).

### Western Blotting

The fresh tumor and adjacent benign tissues were grinded into single-cell suspension and washed twice with ice-cold PBS. The harvested cells were then lysed on ice in lysis buffer for 30 min and centrifuged at 12,000 × *g* for 5 min. Supernatants were collected and the protein concentrations were determined using BCA protein assay kit (Solarbio, China) as suggested by the manufacturer. The same amount of total proteins isolated from cells were resolved by 10% SDS-PAGE and transferred onto polyvinylidene difluoride membranes (Millipore, USA). The membranes were blocked for 1 h and then incubated with monoclonal mouse anti-TLR4 antibody (1:250, Abcam, Cambridge, MA, USA, clone 76B357.1, cat No. ab22048) overnight and then HRP-conjugated secondary antibodies for 1 h, respectively. Signal was visualized using ECL Substrates (Amersham, UK). GAPDH was used as an endogenous protein for normalization.

### Statistics

Statistical Package for the Social Sciences version 22.0 (IBM Corp., USA) and Graphpad Prism 5.0 (GraphPad, USA) were applied to perform all the statistical analysis and drew the figures. The Kolmogorov–Smirnov test was used to assess normality of data. Numerical data were compared using 2-sample *t*-test, paired *t*-test, or Wilcoxon rank sum test. The categorical variables were tested by Fisher’s exact test or Person chi-square test. The correlations between TLR4/PD-L1 expression and between cancer tissues expressed TLR4/serum sTLR4 were assessed using Spearman’s correlation analysis. OS and DFS were determined by the Kaplan-Meier method and compared using log-rank test. Multivariable analysis of the independent factors associated with survival was performed by the Cox proportional hazard model. A two tailed *P*-value < 0.05 was considered statistically significant.

## Results

### High Expression Level of TLR4 in Lung Carcinoma

**Figure 1:**
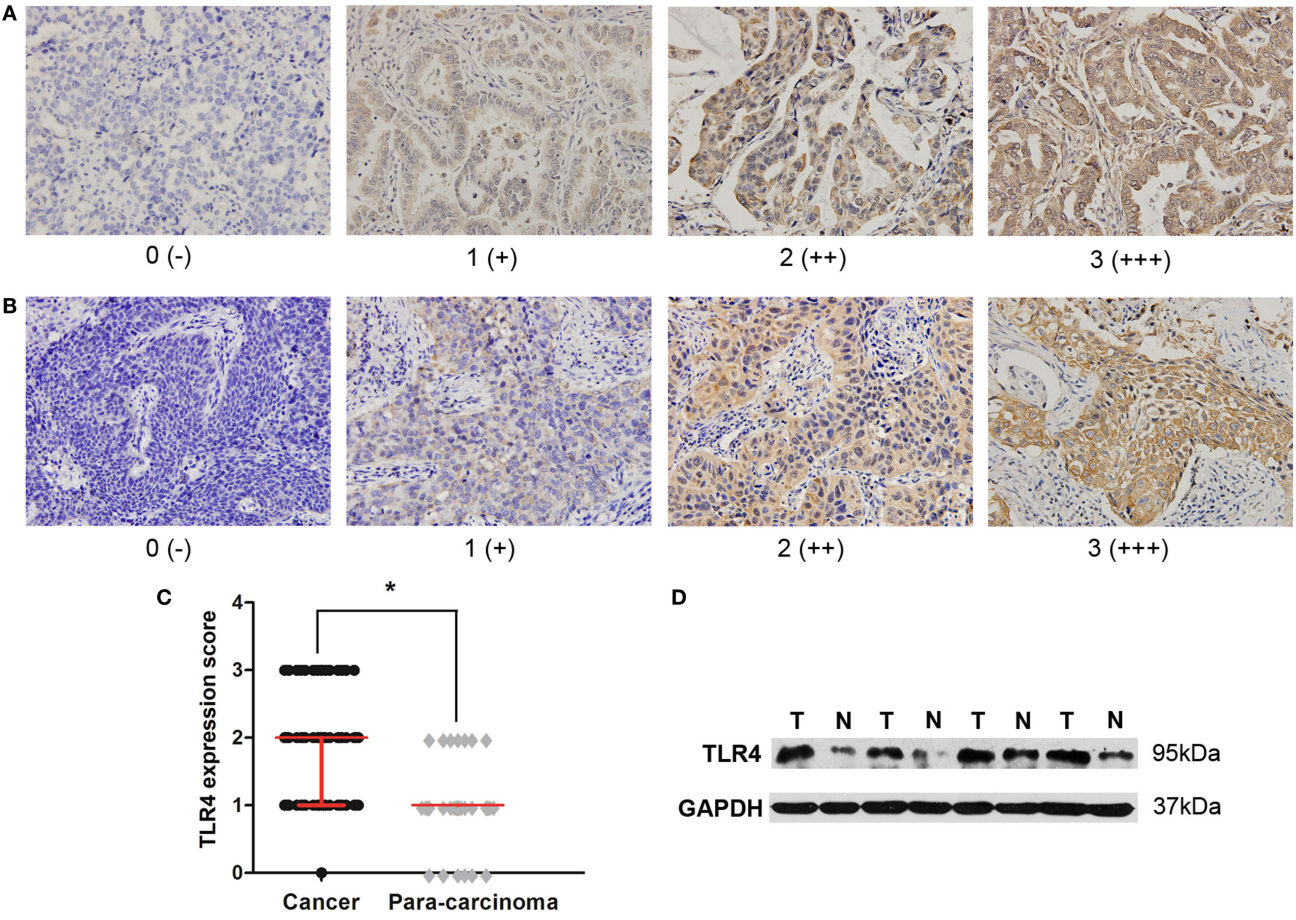
**High expression level of toll-like receptor 4 (TLR4) in non-small cell lung cancer (NSCLC) tissues**. **(A)** Typical immunohistochemical staining of TLR4 in lung adenocarcinoma. Scores 0, 1, 2, and 3 represent negative (−), weak positive (+), moderate positive (++), and strong positive (+++) expression, respectively. **(B)** Typical immunohistochemical staining of TLR4 in lung squamous cell carcinoma. Scores 0, 1, 2, and 3 represent negative (−), weak positive (+), moderate positive (++), and strong positive (+++) expression, respectively. **(C)** Comparison of TLR4 expression level between cancer tissue and para-cancer tissue in 60 patients with NSCLC. TLR4 was expressed at a significantly higher level in cancer cells than in corresponding para-carcinoma cells, as the result of Wilcoxon rank sum test. *P < 0.05. **(D)** Western blotting analysis was further performed to confirm the lower expression of TLR4 in four matched normal tissues (N) compared with tumor tissues (T).

To determine the expression level of TLR4 in lung cancers, immunohistochemical staining with antibody against TLR4 were performed to 126 cases of NSCLC tissues and 60 para-carcinoma tissues. They were scored as 0, 1, 2, and 3 by two pathologists according to the staining intensity and percentage of cell stained. Moreover, scores 0 and 1 were defined as low expression, and scores 2 and 3 were defined as high expression. Positive TLR4 immunostaining was mainly observed in the cytomembrane and cytoplasm of carcinoma cells (Figures 1A,B). From the comparison between cancer samples and their corresponding para-cancer samples of 60 patients, we found that the expression score of cancer tissue was significantly higher than para-cancer tissue (Figure 1C). To further confirm these observations, Western blot assay was done using four paired tumor tissue specimens and matched normal tissues with known levels of TLR4 expression by immunohistochemical staining (Figure 1D).

The correlation of TLR4 expression with clinicopathological characteristics are shown in Table 1. Elevated TLR4 expression was significantly associated with histological type (adenocarcinoma higher than squamous cell carcinoma, *P* = 0.041), increased clinical TNM stage (*P* < 0.001), and presence of lymphatic invasion (*P* < 0.001). However, no statistical relationship was found between TLR4 expression and characteristics such as age, gender, smoking status, tumor size, and SUVmax (median 9.4, and patients were then divided into ≤9.4 group and >9.4 group).

**Table 1:** **Association between TLR4 and PD-L1 expression level and clinicopathologic variables in patients with non-small cell lung cancer**.

	Cases, *n* (%)	TLR4			PD-L1		
		Low	High	*P*-value*	Low	High	*P*-value^#^
**Age (years)**							
<60	50 (40)	20	30	0.930	31	19	0.367
≥60	76 (60)	31	45		53	23	
**Gender**							
Male	70 (55)	30	40	0.543	46	24	0.800
Female	56 (45)	21	35		38	18	
**Smoking status**							
Never smoke	65 (52)	24	41	0.402	42	23	0.614
Smoke	61 (48)	27	34		42	19	
**Histological type**							
SCC	39 (31)	21	18	0.041	23	16	0.220
ADC	87 (69)	30	57		61	26	
**TNM stage**							
I	31 (25)	25	6	<0.001	22	9	0.601
II	34 (27)	12	22		24	10	
III	61 (48)	14	47		38	23	
**Tumor size**							
≤3 cm	77 (61)	36	41	0.072	52	25	0.796
>3 cm	49 (39)	15	34		32	17	
**Lymphatic invasion**							
Negative	47 (37)	30	17	<0.001	38	9	0.009
Positive	79 (63)	21	58		46	33	
**SUVmax**							
≤9.4	62 (49)	26	36	0.743	48	14	0.012
>9.4	64 (51)	25	39		36	28	

*TLR4, toll-like receptor 4; PD-L1, programmed cell death ligand 1; SCC, squamous cell carcinoma; ADC, adenocarcinoma; SUVmax, the maximum standardized uptake value*.

*Pearson chi-square test was used to analyze the above data*.

*P-value refers to the comparison between TLR4 low and high patients, while ^#^P-value refers to the comparison between PD-L1 low and high patients.

### TLR4 Overexpression Indicates a Poor Prognosis in Patients with NSCLC

The average follow-up time was 42 months (median, 36 months; ranging from 12 to 89 months). At the end of follow-up, there were 31 deaths and 95 survivals. The 1-, 3-, and 5-year OS rate of the whole patients was 91, 77, and 56%, respectively, and the corresponding DFS rate was 84, 75, and 50%, respectively. Univariate analysis demonstrated that the OS was significantly associated with smoking status (*P* = 0.033), TNM status (*P* = 0.014), tumor size (*P* = 0.002), SUVmax (*P* = 0.002), and TLR4 expression (*P* = 0.001), while DFS was directly influenced by TNM stage (*P* = 0.020), tumor size (*P* = 0.003), SUVmax (*P* = 0.002), and TLR4 expression (*P* = 0.003). Multivariate analysis showed that expression level of TLR4, tumor size, and SUVmax were independent prognostic factors for OS, and TLR4 expression level and SUVmax were independent prognostic factors for DFS (all *P* < 0.05, Tables 2 and 3).

**Table 2:** **Univariate and multivariate analysis of clinicopathologic factors in NSCLC patients with respect to OS**.

	Cases, *n* (%)	Univariate analysis		Multivariate analysis	
		Survival time (months)	*P*-value	HR (95% CI)	*P*-value
**Age (years)**					
<60	50 (40)	75.71 ± 4.24	0.255		
≥60	76 (60)	57.86 ± 4.22			
**Gender**					
Male	70 (55)	67.25 ± 4.59	0.538		
Female	56 (45)	60.37 ± 5.48			
**Smoking status**					
Never smoke	65 (52)	76.61 ± 4.47	0.033	1.484 (0.593–3.714)	0.400
Smoke	61 (48)	58.53 ± 5.25			
**Histological type**					
SCC	39 (31)	69.39 ± 4.40	0.150		
ADC	87 (69)	52.73 ± 3.85			
**TNM stage**					
I–II	65 (52)	73.60 ± 5.31	0.014	1.137 (0.315–4.105)	0.845
III	61 (48)	54.86 ± 3.98			
**Tumor size**					
≤3 cm	77 (61)	68.79 ± 4.74	0.002	2.695 (1.037–7.509)	0.041
>3 cm	49 (39)	49.16 ± 3.93			
**Lymphatic invasion**					
Negative	47 (37)	68.38 ± 4.35	0.508		
Positive	79 (63)	57.38 ± 2.86			
**SUVmax**					
≤9.4	62 (49)	73.90 ± 4.84	0.002	2.548 (0.979–6.125)	0.046
>9.4	64 (51)	50.16 ± 3.29			
**TLR4**					
Low	51 (40)	72.53 ± 4.78	0.001	3.192 (1.177–8.657)	0.023
High	75 (60)	53.43 ± 4.07			
**PD-L1**					
Low	84 (67)	67.62 ± 4.53	0.212		
High	42 (33)	50.26 ± 3.09			

*TLR4, toll-like receptor 4; PD-L1, programmed cell death ligand 1; SCC, squamous cell carcinoma; ADC, adenocarcinoma; SUVmax, the maximum standardized uptake value*.

*Overall survival (OS) time was presented as mean ± SEM and was determined by the Kaplan-Meier method with log-rank test. Multivariable analysis of the independent factors was performed by the Cox proportional hazard model*.

**Table 3:** **Univariate and multivariate analysis of clinicopathologic factors in NSCLC patients with respect to DFS**.

	Cases, *n* (%)	Univariate analysis		Multivariate analysis	
		Survival time (months)	*P*-value	HR (95% CI)	*P*-value
**Age (years)**					
<60	50 (40)	74.02 ± 4.75	0.212		
≥60	76 (60)	51.85 ± 3.62			
**Gender**					
Male	70 (55)	64.21 ± 5.10	0.529		
Female	56 (45)	52.79 ± 2.81			
**Smoking status**					
Never smoke	65 (52)	60.88 ± 3.97	0.088		
Smoke	61 (48)	58.35 ± 5.77			
**Histological type**					
SCC	39 (31)	68.91 ± 4.95	0.176		
ADC	87 (69)	49.65 ± 4.51			
**TNM stage**					
I–II	65 (52)	71.22 ± 4.55	0.020	1.093 (0.302–3.952)	0.803
III	61 (48)	50.93 ± 4.13			
**Tumor size**					
≤3 cm	77 (61)	67.80 ± 5.56	0.003	2.016 (0.831–4.890)	0.121
>3 cm	49 (39)	45.87 ± 4.46			
**Lymphatic invasion**					
Negative	47 (37)	66.24 ± 5.13	0.307		
Positive	79 (63)	56.26 ± 3.07			
**SUVmax**					
≤9.4	62 (49)	73.78 ± 5.79	0.002	2.587 (0.980–6.162)	0.045
>9.4	64 (51)	46.72 ± 3.79			
**TLR4**					
Low	51 (40)	72.42 ± 5.46	0.003	3.281 (1.199–8.977)	0.021
High	75 (60)	50.07 ± 4.65			
**PD-L1**					
Low	84 (67)	66.86 ± 5.21	0.205		
High	42 (33)	47.48 ± 3.76			

*TLR4, toll-like receptor 4; PD-L1, programmed cell death ligand 1; SCC, squamous cell carcinoma; ADC, adenocarcinoma; SUVmax, the maximum standardized uptake value*.

*Disease-free survival (DFS) time was presented as mean ± SEM and was determined by the Kaplan-Meier method with log-rank test. Multivariable analysis of the independent factors was performed by the Cox proportional hazard model*.

**Figure 2:**
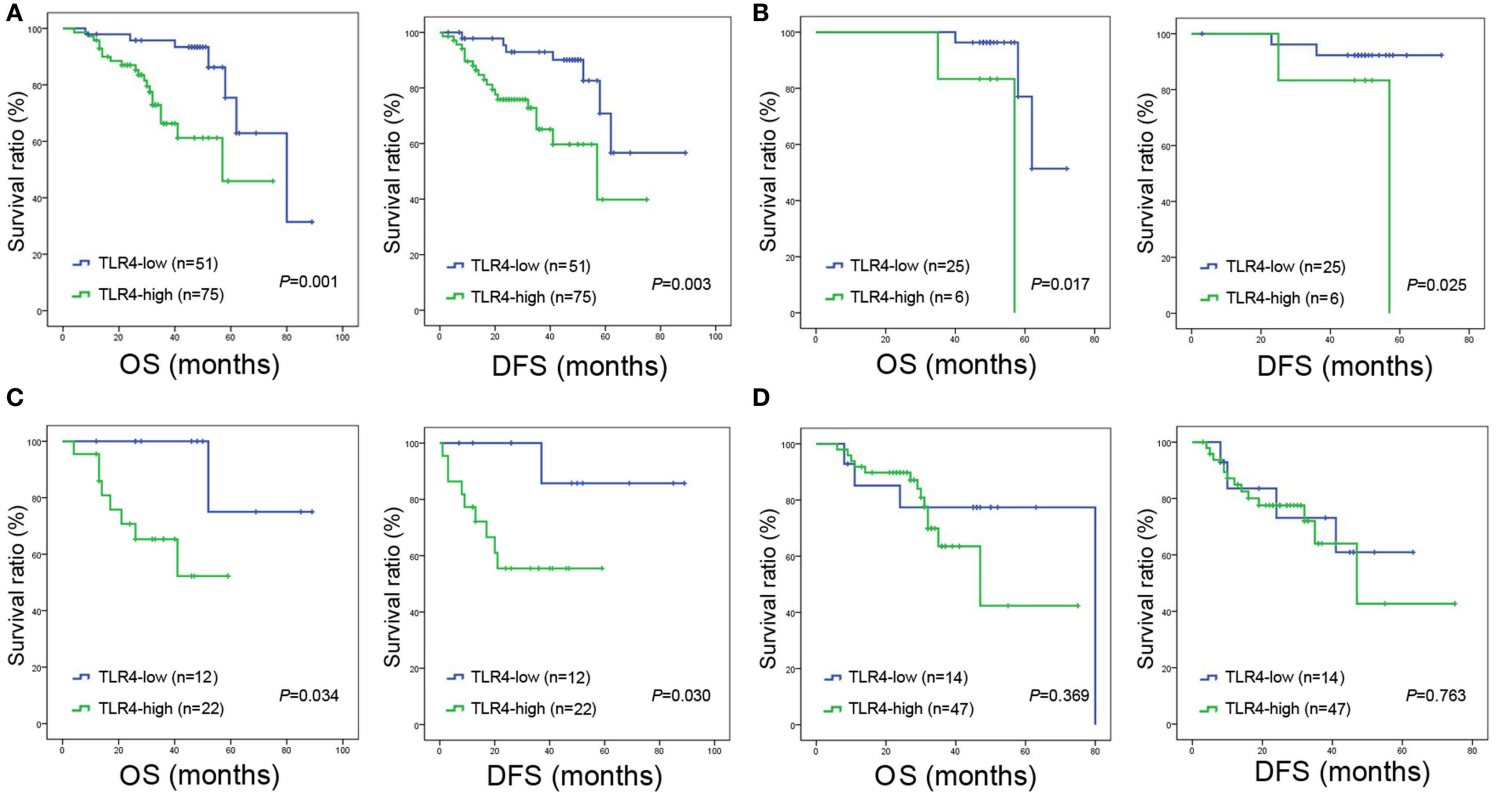
**Overall survival (OS) and disease-free survival (DFS) curves of NSCLC patients according to cancer expressed toll-like receptor 4 (TLR4) levels**. **(A)** All the enrolled patients with high expression of TLR4 were significantly associated with poor OS (*P* = 0.001) and DFS (*P* = 0.003). **(B)** Significantly poor OS (*P* = 0.017) and DFS (*P* = 0.025) in patients with TNM stage I and expressing high level of TLR4. **(C)** Significantly poor OS (*P* = 0.034) and DFS (*P* = 0.030) in patients with TNM stage II and expressing high level of TLR4. **(D)** For stage III, the difference of OS (*P* = 0.369) and DFS (*P* = 0.763) between TLR4 high expression group and TLR4 low expression group is not significant. In panels **(A–D)**, OS and DFS were assessed by the Kaplan-Meier method and compared using log-rank test.

The 1, 3, and 5-year OS rate for all enrolled patients with high TLR4 expression was 87, 63, and 51%, respectively, and 98, 93, and 65 for patients with low TLR4 expression (*P* = 0.001, Figure 2A). The 1, 3, and 5-year DFS rate for patients with high TLR4 was 76, 63, and 49%, respectively, and 95, 90, and 56% for patients with low TLR4 expression (*P* = 0.003, Figure 2A). These all indicated that TLR4 overexpression led to significantly worse prognosis postoperatively. Since TLR4 expression was associated with TNM stage, we further performed survival analysis in patients with separate clinical stage. For stage I and II, patients with high level of TLR4 expression had shorter OS and DFS time than those with low level of TLR4 expression (all *P* < 0.05, Figures 2B,C). For stage III, however, the difference of survival time between TLR4 high expression group and TLR4 low expression group was not significant (*P*_OS_ = 0.369, *P*_DFS_ = 0.763, Figure 2D).

### TLR4 Expression Level in Cancer Samples Is Inversely Correlated with Serum sTLR4 Level in Patients with Early-Stage NSCLC

**Figure 3:**
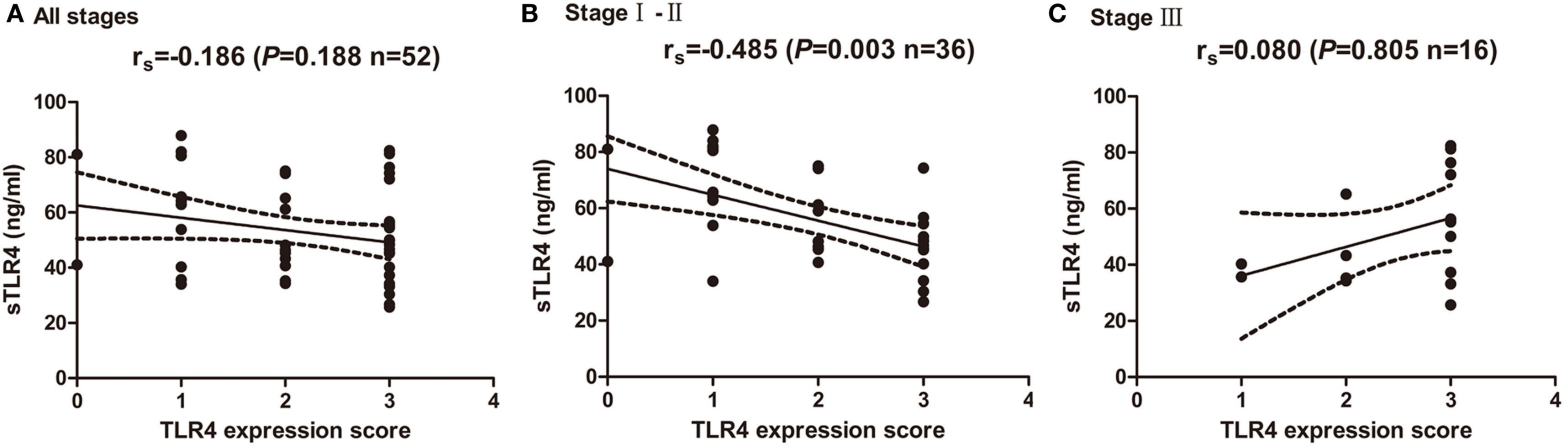
**Correlation of cancer expressed toll-like receptor 4 (TLR4) level and serum soluble TLR4 (sTLR4) level**. **(A)** No significant correlation was found between cancer expressed TLR4 and serum sTLR4 level in all the enrolled patients (*r*_s_ = −0.186, *P* = 0.188). **(B)** TLR4 expression level in cancer tissues is inversely associated with serums sTLR4 level in patients with TNM stage I and II NSCLC (*r*_s_ = −0.485, *P* = 0.003); **(C)** no significant correlation was found between cancer expressed TLR4 and serum sTLR4 level in stage III patients (*r*_s_ = 0.080, *P* = 0.805). Spearman’s correlation analysis was used to determine the above correlations.

The blood samples from 52 NSCLC patients before any treatment for cancer were also obtained and levels of sTLR4 in serum were measured by ELISA. The mean level of sTLR4 is 54.75 ± 18.48 ng/mL. To investigate the relationship of cancer cells expressed TLR4 level and their corresponding serum sTLR4 level, correlation analysis was performed at all stage patients and at different TNM stage subgroups (Figures 3A–C). The results showed that the TLR4 expression level in cancer tissues is inversely associated with serums sTLR4 level in patients with TNM stage I and II NSCLC (*r* = −0.485, *P* = 0.003); nevertheless, this correlation is not obvious in the analysis among all stage patients (*r* = −0.186, *P* = 0.188) and in stage III patients (*r* = 0.080, *P* = 0.805).

### TLR4 Expression Level Is Positively Correlated with the PD-L1 Expression Level in NSCLC

**Figure 4:**
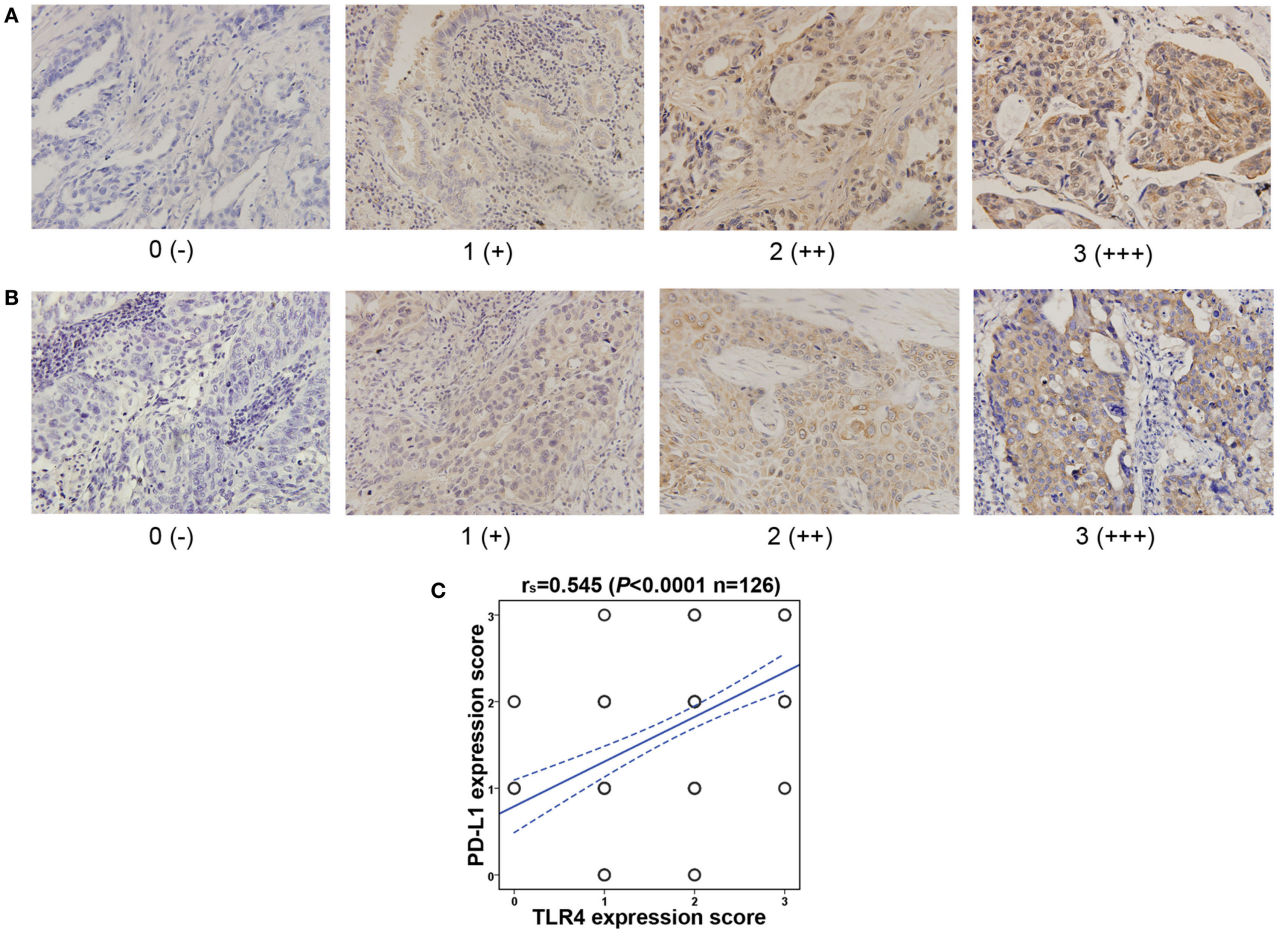
**Toll-like receptor 4 (TLR4) expression level is positively correlated with the programmed cell death ligand 1 (PD-L1) expression level in NSCLC**. **(A)** Representative immunohistochemical staining of PD-L1 in lung adenocarcinoma. Scores 0, 1, 2, and 3 represent negative (−), weak positive (+), moderate positive (++), and strong positive (+++) expression, respectively. **(B)** Typical immunohistochemical staining of PD-L1 in lung squamous cell carcinoma. Scores 0, 1, 2, and 3 represent negative (−), weak positive (+), moderate positive (++), and strong positive (+++) expression, respectively. **(C)** Correlation between cancer tissues expressed TLR4 and PD-L1. TLR4 expression level is positively associated with PD-L1 level, tested by Spearman’s correlation analysis (*r*_s_ = 0.545, *P* < 0.0001).

The level of cancer expressed PD-L1 was also detected by IHC with specific antibody and scored as 0, 1, 2, and 3 representing negative, weak positive, moderate positive, and strong positive expression, respectively, according to the staining intensity and proportion (Figures 4A,B). As shown in Table 1, the expression level of PD-L1 was significantly influenced by lymphatic invasion (*P* = 0.009) and SUVmax (*P* = 0.012), but not by other clinicopathological variables like age, gender, smoking status, histological type, TNM stage, and tumor size (all *P* > 0.05). What is more, univariate and multivariate analysis indicated that the PD-L1 expression level was not associated with the prognostic survival after surgery (*P*_OS_ = 0.212, *P*_DFS_ = 0.205).

The correlation between cancer tissue expressed TLR4 and PD-L1 were then observed. As shown in Figure 4C, the TLR4 expression level was significantly associated with PD-L1 level (*r* = 0.545, *P* < 0.0001). Patients with TLR4 overexpression often led to higher expression of PD-L1.

## Discussion

Lung cancer, most of which can be pathologically diagnosed as NSCLC, is the leading cause of cancer death in China, regardless of gender or ethnicity. It is also the leading cause of cancer-related mortality among men and the second leading cause of cancer death among women worldwide (23). Despite improvement in operation techniques and novel treatment including gene mutation-targeted therapy and checkpoint inhibitor immunotherapy, the prognosis of lung cancer patients remains poor, with an average 5-year survival ratio less than 15% (24, 25). Thus, explorations of crucial mediators and underlying molecular mechanisms involved in cancer growth, metastasis, drug resistance, and recurrence after curative surgical resection are of urgent necessity (26, 27). Recently, accumulating studies indicate that inflammatory mediators and receptors of the tumor microenvironment may play an important role either in lung cancer establishment or in progression and the tumor microenvironment could be characterized by a process of chronic inflammation (28). For lung cancer patients receiving surgical resection, surgery-related systemic inflammation and perioperative pulmonary infection of Gram-negative bacteria could significantly reduce the 5-year OS and 5-year cancer-specific survival (29, 30).

TLR4 recognizes PAMPs like LPS and danger-associated molecular patterns (DAMPs), mainly endogenous ligands such as high-mobility group box 1, heat shock protein 80, and was crucial in the activation of the immune system (31). The significance of cancer expressed TLR4 in cancer-related inflammation has also been outlined. TLR4 overexpression has been detected in various tumor cell lines and could predict the tumor growth or recurrence in gastric tumors, oral tongue squamous cell carcinoma and prostate cancer recurrence (7, 8, 32). For lung cancer, previous research showed that TLR4 were more strongly expressed in lung cancer tissue than para-cancer tissue and was positively correlated with the differentiation degree of tumor cells (10, 11). However, the relationship of TLR4 expression level and survival status of lung cancer patients was not studied. In the present work, we further demonstrated that cancer tissue expressed TLR4 level was negatively associated with the OS and DFS in enrolled patients, and more specifically, in TNM stage I and II patients receiving complete pulmonary resection and systematic lymph node dissection. DAMPs and PAMPs from infection-related inflammatory, tumor-associated inflammatory, or antitumor therapy-induced inflammatory could upregulate the TLR4 expression and activate TLR4 signaling in tumor cells and contribute tumor pathogenesis and progression in a direct or indirect way (33–35). Underlying mechanisms of TLR4 triggering may include the NADPH oxidase 1-dependent ROS pathway, TLR4/ROS/microRNA-21 pathway, mitogen-activated protein kinase (MAPK) and nuclear factor kappa beta (NFκB) pathway, and autophagy of cancer cells to facilitate production of pro-inflammatory and immunosuppressive cytokines, chemokines, and matrix metallopeptidase such as IL-6, IL-8, transformation growth factor-β, vascular endothelial growth factor, chemokine (c-c motif) ligand 2, chemokine (c-c motif) ligand 20, MMP2, and so on (34, 36, 37).

In our previous study, we have investigated the clinical significance of serum sTLR4 in NSCLC and found that low sTLR4 was identified as a prognostic marker for poor survival of early-stage NSCLC patients who received surgical resection. In the present study, we further proved the predicting role of lung cancer cell expressed TLR4 and observed an inverse correlation with serum sTLR4 level in patients with TNM stage I and II NSCLC. The mouse model showed that the sTLR4 was encoded by the alternatively spliced mRNA (38). Aside from competing for the TLR4 ligand binding, sTLR4 could also directly combine and block the membrane synthesizing TLR4 or CD14, thus inhibiting intracellular TLR4 signaling and dampening the stimulating expression of TLR4 on membrane by its ligands (39). What need to be noted is that in stage III lung cancer, the predicting role and the relationship between sTLR4 and cancer cell expressed TLR4 with patients’ survival was not obvious. This means the tumor microenvironment in advanced-stage NSCLC may differ from early-stage NSCLC and could explain the complexity of treatment.

In this study, we also evaluated the expression of PD-L1 in NSCLC by IHC. As a checkpoint receptor, tumor-associated PD-L1 has been shown to inhibit tumor-specific T cell-mediated immunity and cytotoxic T lymphocytes (CTL)-mediated lysis by interacting with PD-1 and other potential unknown receptor on T cells to induce T cell apoptosis and impair cytokine production (12–14, 40). Preliminary data suggested that tumor PD-L1 protein expression using IHC may predict clinical response to PD-1/PD-L1 directed therapy (41, 42). However, there are limited data on the prevalence and the prognostic role of PD-L1 expression in NSCLC. Several previous studies have demonstrated conflicting results varying from poor prognosis of PD-L1 overexpression to no prognostic significance and to better outcome (43–46). In the present study, though PD-L1 expression is associated with lymphatic invasion and SUVmax, no statistical correlation with postoperative survival was observed. These data are likely confounded by the small, heterogeneous sample size, as well as the distinct definition of PD-L1 positivity or grading by IHC. What is more, the PD-L1 expression could dynamically increase in response to the interferon-γ (INF-γ) released from CD8^+^ T cells in tumor microenvironment as an adaptive immune resistance (47), and this seems to be more common than the constitutive expression of PD-L1 in most cancer histologies (48, 49). Therefore, the prognostic value of PD-L1 expression would be compromised by the status of tumor-infiltrating lymphocytes (TIL). And now the classification of tumor microenvironment based on the presence or absence of TIL and PD-L1 expression has already been established (50).

In the present work, we found that TLR4 expression was positively correlated with PD-L1 expression. This is in accordance with the recent cellular and animal studies which indicated that PD-L1 expression in cancer cells could be upregulated by TLRs, especially TLR4 stimulation aside from the inducibility of INF-γ. In acute myeloid leukemia, PD-L1 protection of blasts from cytotoxic T cells is induced by TLR stimulation and INF-γ and can be reversed using MAPK/extracellular-signal-regulated kinases (Erk) kinase (MEK) inhibitors (15). Plasma cells from multiple myeloma patients express PD-L1 and increase expression after stimulation with IFN-γ and TLR ligands via a myeloid differentiation primary response gene 88/tumor necrosis factor receptor associated factor 6 (MYD88/TRAF6)-, MEK-dependent pathway (51). Activation of TLR4 signaling in bladder cancer cells induces tumor-associated PD-L1 expression via activation of Erk and c-Jun N-terminal kinase pathways and protects tumor from CTLs killing (52, 53). Human tumors have shown significant correlations between PD-L1 expression and levels of T cell infiltration (22, 48, 54). Similarly, the inducible expression of PD-L1 by TLR4 could explain the correlated expression of PD-L1 and TLR4 in NSCLC in the present study, and this could be one potential mechanism of PD-L1 overexpression in TILs negative tumor microenvironment except for the genetic alternations in intrinsic immune resistance.

There were two limitations in this study. First, we investigated the expression of TLR4 and PD-L1 only in patients with TNM stage I to IIIa patients having opportunity to receive surgical therapy, for those advanced NSCLC patients, the data were unavailable, and needs to be further studied. In addition, the consensus on defining a threshold for positive PD-L1 labeling on biopsy tissue samples by IHC still remains a challenge. Therefore, alternative tests like RNA-based assay could be performed to verify the role of PD-L1 expression in NSCLC and its relationship with TLR4.

Taken together, we proved that TLR4 was strongly expressed in lung cancer tissues and predicted poor prognosis of patients with NSCLC. What is more, cancer expressed TLR4 was inversely correlated with serum sTLR4 level and was positively correlated with PD-L1 expression in lung cancer tissues. To our knowledge, this is the first time to associate TLR4 with lung cancer patients’ survival status and to explore correlation between TLR4 and PD-L1 expression. Thus, specific treatment targeting TLR4 might have a synergistic effect with PD-L1 blockage. All the current findings indicated that TLR4 was a valuable predicting marker that might provide help for clinicians to design effective therapeutic modality against lung cancer.

## Ethics Statement

This study was carried out in accordance with the recommendations of the biomedical research guidelines involving human participants established by the National Health and Family Planning Commission of China. The Ethical Committee of TMUCIH approved this study, and written informed consent was obtained from each subject in accordance with the Declaration of Helsinki.

## Author Contributions

XR and KW designed the study protocol. KW and JW wrote the manuscript. KW, NZ, and JW performed the experiments. FW and FY analyzed the collected data and revised the manuscript.

## Conflict of Interest Statement

The authors declare that the research was conducted in the absence of any commercial or financial relationships that could be construed as a potential conflict of interest.
